# Trends in hepatitis B notification rates in Guangzhou, China, between 2009 and 2020: an epidemiological study

**DOI:** 10.1186/s12879-022-07690-y

**Published:** 2022-12-07

**Authors:** Wei Liu, Zhiqiang Dong, Wensui Hu, Ke Li, Lili Sun, Jianrong Hou, Shijie Jia, Yuan Liu

**Affiliations:** 1 Operations Management Section, Guangzhou Center for Disease Control and Prevention, No. 1, Qide Road, Jiahe, Baiyun District, Guangzhou, 510440 China; 2 Department of Traditional Chinese Medicine, Guangzhou Eighth People’s Hospital Guangzhou Medical University, No.627, Dongfeng East Road, Yuexiu District, Guangzhou, 510060 China

**Keywords:** Hepatitis B, Epidemiology, Temporal trends, Joinpoint regression

## Abstract

**Background:**

Although the prevalence of hepatitis B in Guangzhou, China, is high, the epidemiological trends are not well-documented. We aimed to analyse newly reported hepatitis B cases in Guangzhou between 2009 and 2020 to explore the epidemiological trends and provide insights for the development of control measures.

**Methods:**

Information on the population and new cases of hepatitis B in Guangzhou between 2009 and 2020 was obtained from the China Information System for Disease Control and Prevention, which was used to calculate the annual notification rates of hepatitis B by sex, age group (0–9; 10–19; 20–29; 30–39; 40–49; 50–59; ≥ 60 years), and location (urban or rural). Joinpoint regression analysis was used to analyse the temporal trends and calculate the average annual percentage change (AAPC) and annual percentage change (APC) for each identified trend line segment.

**Results:**

Between 2009 and 2020, 287,034 new cases of hepatitis B were cumulatively reported. The average annual notification rate was 181.13/100,000, and the notification rate showed a long-term downward trend during the period 2009–2020, with an annual decrease of 6.30% (APC − 6.30%; 95% CI − 7.56 to − 5.02%). Men had a significantly higher notification rate than women; however, the sex ratio decreased from a maximum of 2.34 in 2010 to a minimum of 1.54 in 2020. A downward trend in the notification rate was observed in urban areas and an upward trend was observed in rural areas, with an increase in the rural/urban ratio from 0.46 in 2012 to 1.57 in 2020. The notification rate for all age groups showed a decreasing trend from 2009, with the exception of the 50–59 years and ≥ 60 years groups, whose notification rates began to decrease from 2014 and 2015, respectively.

**Conclusions:**

Although the overall notification rate of hepatitis B in Guangzhou decreased annually, it remained high. Further, in rural areas, the notification rate has been increasing, and effective measures should be taken to control hepatitis B infection in Guangzhou.

## Background

Hepatitis B is caused by infection with the hepatitis B virus (HBV) and has become a major global public health problem [1, 2]. The latest data from the World Health Organization (WHO) revealed that 296 million people were living with chronic hepatitis B (CHB), and hepatitis B resulted in an estimated 820,000 deaths worldwide in 2019 [3]. Several studies have shown that the prevalence of hepatitis B varies by geographical region, with infection rates of < 2% in the United States and Western Europe, 2–8% in the Mediterranean and South America, and up to 20% in Asia and Africa [4–6]. In addition, significant sex, age, race, and urban–rural differences in HBV infection are frequently reported, although these reports are not consistent [5, 7, 8]. For example, a meta-analysis of Chinese populations reported that the prevalence of HBV infection was higher in men than in women and in rural rather than urban areas [7], but an Iranian study found that the rate of infection was higher in the urban areas [8].

Hepatitis B virus is highly contagious and transmitted primarily via perinatal and percutaneous routes and sexual exposure as well as by close person-to-person contact, presumably via open cuts and sores [9]. HBV is highly resistant, surviving up to 30 days at room temperature on dry surfaces [10]. Individuals with CHB infection are at an increased risk of developing HBV-related liver diseases, such as liver cirrhosis and hepatocellular carcinoma (HCC) [11, 12]. Studies have shown that 15–40% of people with CHB infection develop cirrhosis, HCC, or liver failure, and 25% die prematurely from these complications [9, 13–15].

**Fig. 1:**
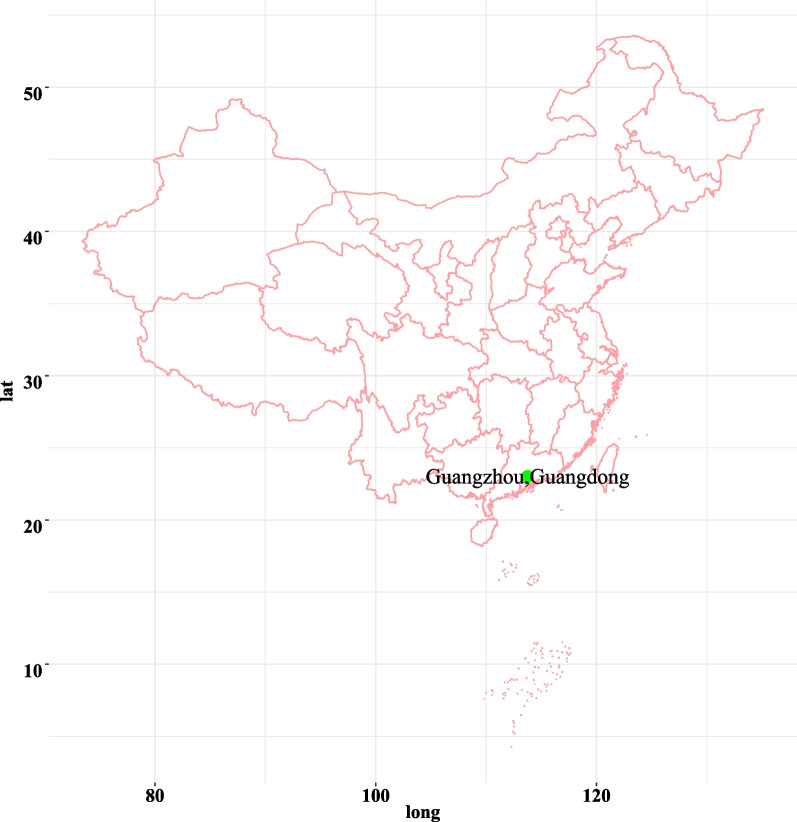
Geographical location of the study area (Guangzhou, Guangdong)

In China, a national epidemiological survey reported that the positive rate of hepatitis B surface antigen (HBsAg) in the Chinese population was 7.20% in 2006. However, the positive rate in Guangdong Province was 11.10% during the same period, which was much higher than the national average [16]. Guangzhou is the capital of Guangdong province, located in the south of China (Fig. 1), with a resident population of 18.7 million in 2020, a developed economy, and frequent movement of people. The most recent large-scale population-based epidemiological survey of hepatitis B in 2018 demonstrated that the rate of HBsAg positivity in the Guangzhou population was 12.45% [17], which was higher than the national rate and the provincial rate in 2006 [16, 18]. Unfortunately, few studies have investigated the changing trends in hepatitis B infection in Guangzhou.

This study aimed to describe the trends in the reported prevalence of HBV infection in Guangzhou between 2009 and 2020 in order to provide a reference for the government to develop strategies and measures for the prevention of hepatitis B infection.

## Methods

### Data sources

Information on the number of hepatitis B infection cases and the population, stratified by sex, age, and location, was obtained from the China Information System for Disease Control and Prevention (CISDCP). The CISDCP is a web-based infectious disease reporting system covering 40 notifiable diseases (COVID-19 was included in January 2020). The system was established in 2004, requiring medical institutions and disease prevention and control institutions at all levels nationwide to report to the system in the first instance when the above 40 notifiable diseases were detected. The system also provides a standardized case reporting form for collecting demographic and diagnostic information for each person afflicted by reportable diseases [19–22]. Reporting of infectious diseases is the responsibility of the doctor who first identifies an infectious disease, and positive cases found by blood collection agencies during blood testing should also be reported immediately. In addition to reviewing and deleting duplicate cases reported by hospitals on a daily basis, CDCs at all levels also organize several supervisory inspections of the quality of hospital infectious disease reports with health administration departments each year to ensure data quality. Clinically diagnosed and laboratory confirmed cases with an onset date between January 1, 2009 and December 31, 2020 (excluding those not clearly classified as acute or chronic hepatitis B cases) and a current address in Guangzhou were screened as the study population.

All cases were divided by age into seven groups comprised of 0–9, 10–19, 20–29, 30–39, 40–49, 50–59, and ≥ 60 years age groups. Based on socio-economic development, the Zengcheng and Conghua districts were classified as rural areas and the remaining nine districts, such as Yuexiu and Liwan, were classified as urban areas. Notification rates were calculated per 100,000 persons.

### Statistical analysis

We used Excel for data collection, and R software (version 4.1.1) was used for data cleaning and descriptive statistics, such as the hepatitis B notification rate, notification rate ratio of males to females and rural to urban areas, etc., for all age groups during the study period. Joinpoint regression analysis software (version 4.9.0.0. March 2021; Statistical research and applications branch, National Cancer Institute) was used to examine the trends in the notification rates of hepatitis B infection for the whole population and subgroup populations during the study period. A maximum of two joinpoints were permitted based on the number of data points. The Grid Search method and Monte Carlo permutation tests were performed to identify the best-fitting combination of line segments and joinpoints. The average annual percentage change (AAPC) and associated 95% confidence interval (CI) were calculated to measure the direction and magnitude of trends over the full range of time periods. We also calculated the annual percentage change (APC) and 95% CIs of each line segment. A two-sided *p*-value of < 0.05 was considered statistically significant.

## Results

### Overall trend in the hepatitis B notification rate in the whole population

**Fig. 2:**
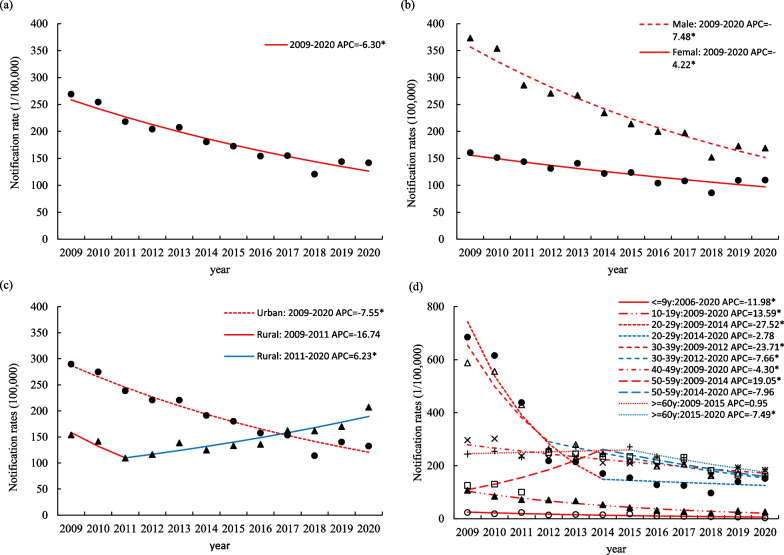
Joinpoint model estimates in Guangzhou during 2006–2020. **a** Trends in the overall notification rate. **b** Trends in the notification rate stratified by sex. **c** Trends in the notification rate stratified by location. **d** Trends in the notification rate stratified by age

Between 2009 and 2020, 287,034 new hepatitis B cases were reported cumulatively in Guangzhou. Among them, 67.96% were men, the median age was 38 years (29–52 years), and the average annual notification rate was 181.13 per 100,000 persons, with the highest in 2009 (269.16 per 100,000) and the lowest in 2018 (120.62 per 100,000). Joinpoint regression analysis showed that the notification rate declined over the whole study period (2009–2020) with no joinpoint (APC − 6.30%; 95% CI − 7.56 to − 5.02%) (Table 1; Fig. 2a).

**Table 1:** Notification rate of hepatitis B in Guangzhou, 2009–2020 (1 per 100,000)

	2009	2010	2011	2012	2013	2014	2015	2016	2017	2018	2019	2020
Overall	269.16	254.55	218.10	204.24	207.45	180.55	172.52	154.11	154.93	120.62	144.01	141.73
Sex												
Male	373.31	353.98	285.90	270.77	267.16	234.30	213.84	200.00	197.43	152.09	172.91	168.93
Female	160.62	151.34	143.89	131.33	140.78	121.99	123.69	104.01	108.11	85.95	109.30	109.59
Male/female	2.32	2.34	1.99	2.06	1.90	1.92	1.73	1.92	1.83	1.77	1.58	1.54
Location												
Rural	153.60	141.47	109.29	116.20	138.58	124.74	133.10	135.88	162.22	161.64	169.81	207.22
Urban	289.72	274.82	238.49	220.91	220.62	191.22	180.06	157.59	153.54	114.01	140.32	132.39
Rural/urban	0.53	0.51	0.46	0.53	0.63	0.65	0.74	0.86	1.06	1.42	1.21	1.57
Age group												
0–9 years	24.79	21.07	31.80	19.77	17.99	16.97	19.23	10.87	10.13	8.23	6.36	3.55
10–19 years	76.78	59.48	46.24	49.13	45.78	37.50	30.98	20.47	17.21	13.98	19.86	16.43
20–29 years	671.98	601.05	427.51	203.56	199.89	158.72	142.07	115.81	110.26	85.20	121.43	131.10
30–39 years	612.20	575.76	437.87	271.50	283.93	247.91	219.20	203.95	215.01	170.35	175.58	165.94
40–49 years	318.37	320.69	255.23	239.43	244.41	217.95	218.32	203.90	212.16	161.84	196.32	185.21
50–59 years	139.61	143.49	109.47	277.95	283.43	269.40	262.46	245.65	255.04	205.10	188.19	181.53
≥ 60 years	244.25	254.05	231.92	264.80	271.36	241.68	271.52	236.00	230.97	174.95	194.47	184.90

### Trends in the hepatitis B notification rate among the subgroups

**Fig. 3:**
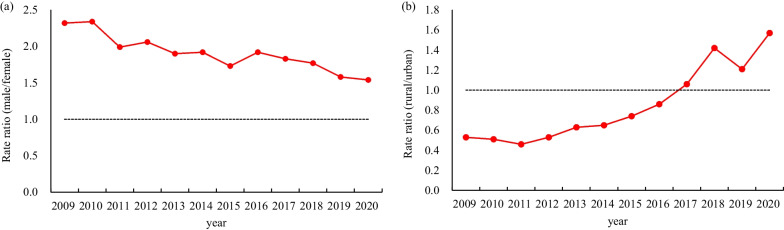
Trends in the notification rate. **a** Trends in the sex ratio. **b** Trends in the location ratio

Overall, the notification rate was significantly higher in men than women, but this gap was reduced annually. The sex ratio (male/female) decreased from a maximum of 2.34 in 2010 to a minimum of 1.54 in 2020 (Fig. 3a). Before 2017, the notification rate of hepatitis B in rural areas was lower than that in urban areas; however, after 2017, the notification rate in rural areas had exceeded that in urban areas and the gap gradually widened. The rural to urban ratio increased from a minimum of 0.46 in 2011 to a maximum of 1.57 in 2020 (Fig. 3b).

Trends in the sex-specific notification rate of hepatitis B are depicted in Fig. 2b. For both men and women, the trend in the hepatitis B notification rate was generally consistent with the overall notification rate, and joinpoint regression analysis showed that no joinpoints were detected. For men, the notification rate declined from 373.31 per 100,000 in 2009 to 168.93 per 100,000 in 2020, with an APC of − 7.48% (APC − 7.48%; 95% CI − 8.69 to − 6.26%). For women, the notification rate declined from 160.62 per 100,000 in 2009 to 109.59 per 100,000 in 2020, with an APC of − 4.22% (APC − 4.22%; 95% CI − 5.72 to − 2.69%).

Figure 2c shows the trends in hepatitis B notification rates in both rural and urban areas. From 2009 to 2020, the notification rate in urban areas showed a decreasing annual trend, with an annual decrease of 7.75% (APC − 7.75%; 95% CI − 8.80 to − 6.33%); in rural areas the notification rate exhibited an overall increasing trend, with an average annual increase of 1.62% (AAPC 1.62%; 95% CI − 2.82 to 6.28%), joinpoint regression analysis showed that after a short-term decrease from 2009 to 2011 (APC − 16.74%; 95% CI − 36.98 to 10.01%), the notification rate began to exhibit a long-term increasing trend (APC 6.23%; 95% CI 3.82 to 8.69%).

The trends in the age-specific notification rate of hepatitis B are depicted in Fig. 2d. Joinpoint regression analysis indicated that no joinpoints were detected in the 0–9 years, 10–19 years and 40–49 years age groups, and the notification rate of hepatitis B showed a decreasing annual trend in all these three age groups, with an annual decrease of 11.98% (APC − 11.98%; 95% CI − 15.96 to − 7.81%), 13.59% (APC − 13.59%; 95% CI − 15.71 to − 11.48%) and 4.30% (APC − 4.30%; 95% CI − 5.75 to − 2.82%), respectivly. Similar trends were observed in the 20–29 years and 30–39 years age groups, both of which showed a rapid decline followed by a slower decline or remained stable. The notification rate in the 20–29 years age group declined at a rate of 27.52% (APC − 27.52%; 95% CI − 35.49 to − 18.57%) per year until 2014, after which it remained stable. The 30–39 years age group maintained a declining trend at a rate of 23.71% (APC − 23.71%; 95% CI − 30.83 to − 15.85%) per year from 2009 to 2012, before maintaining a rate of decline of 9.78% (APC − 9.78%; 95% CI − 5.49 to − 8.11%) until 2020. The notification rate in the 50–59 years age group showed a consistent increase until 2014 (APC 19.05%; 95% CI 1.14 to 40.12%), after which it began to decline, but not significantly. One joinpoint in 2015 were identified in the ≥ 60 years age group, the notification rate exhibited an increasing but not significant trend until 2015, after which it began to decline rapidly (APC − 7.49%; 95% CI − 12.60 to − 2.09%).

## Discussion

Liver disease caused by HBV infection has resulted in a huge disease burden worldwide, and CHB infection is particularly prevalent in Asia, specifically in China [23–26]. HBV is transmitted in a complex and insidious manner; the primary modes of HBV transmission in Guangdong include mother-to-child transmission, family aggregate transmission, traumatic medical practices, and paid blood donation [27, 28]. In 2016, the WHO approved the first global health sector strategy for viral hepatitis, with the goal of eliminating viral hepatitis as a major public health threat by 2030 (reducing new infections by 90% and mortality by 65%) [24].

The present study aimed to describe the trends in the reported prevalence of HBV infection in Guangzhou between 2009 and 2020 in order to provide a reference for the government to develop strategies and measures for the prevention of hepatitis B infection. We found that the notification rate for new hepatitis B cases in Guangzhou decreased from 269.16 per 100,000 in 2009 to 141.73 per 100,000 in 2020, with an annual decrease of 6.30%. A long-term decreasing trend was noted in the notification rate of hepatitis B in Guangzhou, which can likely be attributed to a series of interventions and the introduction of relevant policies by the Chinese government. In 1992, a recombinant vaccine was licensed and introduced nationwide for a fee, requiring one dose of hepatitis B vaccine for all births and two additional doses during infancy, with the goal of interrupting perinatal HBV transmission and providing lifelong HBV protection for new-borns. In 2002, China included the hepatitis B vaccine in its Expanded Program on Immunization (EPI), providing free vaccinations for children aged < 14 years. In addition, between 2009 and 2011, a catch-up campaign was launched for children aged < 15 years, which succeeded in vaccinating approximately 68 million children [26, 29]. Despite the efforts of the government and significant decrease in the notification rate of hepatitis B in Guangzhou, the notification rate of 141.73 per 100,000 in 2020 is still significantly higher than that in certain developed western countries, such as the United States and European Union (EU) countries [30, 31], and Asian countries, including Japan and South Korea [32, 33], and is also higher than the national average of 69.05 per 100,000 in 2014 [34]. Guangzhou is still under tremendous pressure to achieve the 2030 target set by the WHO.

This study highlights a long-term decreasing trend in the notification rates in both sexes. The notification rate is currently significantly higher in men than in women, although this sex difference has decreased from 2.34-fold in 2010 to 1.54-fold in 2020, which is consistent with the reports from EU/European Economic Area countries in 2018 [31]. One of the main reasons for this observation is that variant oestrogen receptors are more highly expressed in male HCC patients than in female patients, leading to speculation that oestrogen may play a role in protection against HBV infection [35]. Moreover, China has implemented universal free mandatory hepatitis B vaccination, thus generating universal resistance in both men and women, which further reduced the sex difference in the prevalence of HBV infection.

In addition, this study also found that the notification rate in urban areas has been declining at an average annual rate of 7.75% since 2009. However, rural areas showed a brief decline from 2009 to 2012, after which it began to increase at an annual rate of 6.23%. The decline in urban morbidity may be attributed to the benefits of a series of government interventions and policies, such as the free EPI for Children aged < 14 years in 2002, and the 2009–2011 vaccine catch-up campaign for children aged < 15 years. The long-term increasing trend in rural areas since 2012 may be attributed to the increasing degree of urban–rural integration with the development of China's economy, which has significantly improved the level of medical care, medical resources, and the convenience of medical care for residents in rural areas; and the gradually increasing living standards, literacy, and awareness of medical care among rural residents. The combination of these factors has contributed to the increase in the diagnostic rate of hepatitis B in rural areas and the rate of medical consultation among the residents, ultimately leading to an annual increase in the notification rate of hepatitis B. The notification rate of hepatitis B in rural areas exceeded that in urban areas for the first time in 2017, and the gap widened annually, reaching 1.57-fold the rate in urban areas by 2020, which is inconsistent with the significantly higher prevalence of acute hepatitis B in urban areas than in rural areas reported in Poland and Norway [36, 37]. Possible explanations are that the prevalence of hepatitis B in these countries is low and many of the new cases are immigrants, who mostly live in urban areas [37, 38].

There was an overall decreasing trend in all age groups except 50–59 years and ≥ 60 years age groups. This may be primarily due to China's vaccination policy and the improvement of medical care, which effectively prevented the spread of hepatitis B transmission. The notification rate in the 50–59 years age group continued to rise until 2014, after which it began to decline, and in the group aged ≥ 60 years, after an increasing but not significant trend until 2015, the notification rate began to decline annually. We are unable to provide an explanation for the trends in the notification rate in these two age groups, and further studies are needed.

Our analysis had several strengths. First, with the continuous improvement of the CISDCP system and the increasingly standardized implementation of infectious disease reporting requirements by medical institutions, the CISDCP system includes almost all new cases of hepatitis B. One study indicated that the overall rate of underreporting of infectious diseases in Guangzhou is 1.17%, and the total rate of underreporting of infectious diseases of category B (to which hepatitis B belongs) is 0.36%; this rate is even lower if only hepatitis B is calculated [39]. Second, we included up to 12 years of data in the analysis, which greatly increased our ability to detect temporal trends and led to more consistent results. However, our study also has several limitations. First, although the underreporting rate of the CISDCP system was low, the consultation rate of hepatitis B patients was not available. It can be speculated from the annual increased notification rate in rural areas that there may still be numerous undiagnosed patients without symptoms in rural areas and that the true incidence of hepatitis B may be markedly higher than that observed in this study. Second, the purpose of this study was to analyze trends in the notification rate of hepatitis B in Guangzhou, and we were unable to quantify exposure to risk factors (such as the standard of living, awareness of access to medical care, and local level of medical care) that may contribute to the change in HBV infection tendencies. However, we believe that these external factors are gradual and continuous processes and should have little effect on the reported incidence in the approaching years.

## Conclusions

The overall notification rate of hepatitis B in Guangzhou is decreasing annually; however, there has been an increasing trend in rural areas in recent years. Effective measures should be taken to control hepatitis B infection in Guangzhou, particularly in rural areas.

## Data Availability

The datasets used and analysed in the current study are available from the corresponding author on reasonable request.

## Abbreviations

HBV: Hepatitis B virus
CHB: Chronic hepatitis B
HCC: Hepatocellular carcinoma
HBsAg: Hepatitis B surface antigen
WHO: World Health Organization
CISDCP: China Information System for Disease Control and Prevention
AAPC: Average annual percentage change
APC: Annual percentage change
CI: Confidence interval
EPI: Expanded Program on Immunization
EU: European Union
